# New antibiotics for bad bugs: where are we?

**DOI:** 10.1186/1476-0711-12-22

**Published:** 2013-08-28

**Authors:** Matteo Bassetti, Maria Merelli, Chiara Temperoni, Augusta Astilean

**Affiliations:** 1 Infectious Diseases Division, Santa Maria Misercordia Hospital, Udine, Italy; 2 Clinica Malattie Infettive, Azienda Ospedaliera Universitaria Santa Maria della Misericordia, Piazzale Santa Maria della Misericordia 15, Udine 33100, Italy

**Keywords:** New antibiotics, Resistance, Bacteria, FDA, EMA

## Abstract

Bacterial resistance to antibiotics is growing up day by day in both community and hospital setting, with a significant impact on the mortality and morbidity rates and the financial burden that is associated. In the last two decades multi drug resistant microorganisms (both hospital- and community-acquired) challenged the scientific groups into developing new antimicrobial compounds that can provide safety in use according to the new regulation, good efficacy patterns, and low resistance profile. In this review we made an evaluation of present data regarding the new classes and the new molecules from already existing classes of antibiotics and the ongoing trends in antimicrobial development. Infectious Diseases Society of America (IDSA) supported a proGram, called “*the ′10 × ´20′ initiative*”, to develop ten new systemic antibacterial drugs within 2020. The microorganisms mainly involved in the resistance process, so called the ESKAPE pathogens (*Enterococcus faecium, Staphylococcus aureus, Klebsiella pneumoniae, Acinetobacter baumanii, Pseudomonas aeruginosa,* and *enterobacteriaceae*) were the main targets. In the era of antimicrobial resistance the new antimicrobial agents like fifth generation cephalosporins, carbapenems, monobactams, β-lactamases inhibitors, aminoglycosides, quinolones, oxazolidones, glycopeptides, and tetracyclines active against Gram-positive pathogens, like vancomycin-resistant *S. aureus* (VRSA) and MRSA, penicillin-resistant streptococci, and vancomycin resistant *Enterococcus* (VRE) but also against highly resistant Gram-negative organisms are more than welcome. Of these compounds some are already approved by official agencies, some are still in study, but the need of new antibiotics still does not cover the increasing prevalence of antibiotic-resistant bacterial infections. Therefore the management of antimicrobial resistance should also include fostering coordinated actions by all stakeholders, creating policy guidance, support for surveillance and technical assistance.

## Introduction

Bacterial resistance to antibiotics is growing up day by day in both community and hospital setting, increasing mortality and morbidity [1].

Nowadays, the continuous development and the spread of bacterial resistances pose some questions about their future and represent a serious threat for their clinical utility, leading to an urgent requirement for new compounds.

Multidrug resistance (MDR) bacteria is defined as non-susceptibility to one or more antimicrobials on three or more antimicrobial classes, while strains that are non-susceptible to all antimicrobials, are classified as extreme drug-resistant strains [2]. MDR bacteria have a significant impact on mortality, hospital stay and associated-costs [3].

 The microorganisms that are mainly involved in the resistance process are the, so called the ESKAPE pathogens (*Enterococcus faecium, Staphylococcus aureus, Klebsiella pneumoniae, Acinetobacter baumanii, Pseudomonas aeruginosa,* and *enterobacteriaceae*) emphasizing their capacity to “escape” from common antibacterial treatments [4].

**Figure 1:**
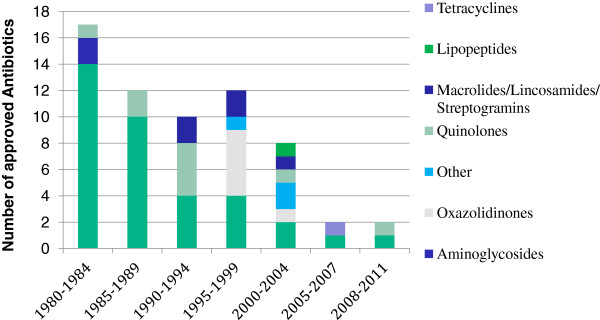
Number of approved antibiotics during the last 30 years.

However despite this scenario, since 2000 only three new classes of antibiotics have been introduced to the market for human use and one of those is limited to topical use (Figure 1) [5]. ‘Innovation gap’ is the expression that has been used to describe the lack of novel structural classes introduced to the antibacterial armamentarium since 1962.

Numerous agencies and professional societies have tried to draw attention to the lack of new antibiotics, especially for MDR Gram-negative pathogens. Since 2004 repeated calls for reinvigorating pharmaceutical investments in antibiotic research and development have been made by the Infectious Diseases Society of America (IDSA) and several other distinguished societies [6].

Recently, IDSA supported a proGram, called “*the ′10 × ′20′ initiative*”, to develop ten new systemic antibacterial drugs within 2020 through the discovery of new drug classes, as well as to find possible new molecules from already existing classes of antibiotics [7].

In this review the new drugs belonging to both old and new classes of antibiotics will be analysed and discussed (Table 1).

**Table 1:** New antibiotics approved and/or in development

**Drug**	**Antibiotic class**	**Spectrum of microbiological activity**	**Main clinical indication**	**Development phase**
**BAL30072**	Monocyclic β-lactam	MDR P. aeruginosa Acinetobacter including metallo-ß-lactamases and enterobacteriaceae	NK	I
**BC-3781**	Pleuromutilin	Gram-positive, including MRSA	cSSSIs	II
**Besifloxacin**	Quinolone	Gram-positive and Gram-negative	ophthalmicinfection	Approved by FDA
**Biapenem**	Cerbpenem	Gram-negative and Gram-positive	RTI, UTI	II
**CB-182,804**	Polimyxin	MDR Gram-negative	NK	I
**Ceftarolinefosamil**	Cephalosporin	Gram-positive	cSSSIs, CAP	Approved by FDA and EMA
**Ceftazidime/Avibactam**	Cephalosporin + betalactamase-inhibitor	MDR *P. aeruginosa*and enterobacteriaceae, excluding metallo-ß-lactamases	cUTI, cSSTI, VAP	III
**Ceftobiprolemedocaril**	Cephalosporin	Gram-positive	cSSTI, hospitalized CAP	III
**Ceftolozane/tazobactam**	Cephalosporin + betalactamase-inhibitor	Gram-negative	cIAI, cUTIs, HAP, VAP	III
**Cethromycin**	Ketolide	Gram-positive and Gram-negative	CAP	III
**Dalbavancin**	Glycopeptide	Gram-positive	cSSTI	III
**Delafloxacin**	Quinolone	Broad-spectrum including fluoroquinolone-resistant MRSA	cSSTI	II
**Doripenem**	Carbapenem	Gram-negative	cUTIs, cIAIs, HAP, VAP	Approved by FDA and EMA
**Eravacycline**	Tetracycline	Gram-negative but not Pseudomonas	cIAI	II
**JNJ-Q2**	Quinolone	Enhanced Gram-positive activity including fluoroquinolone-resistance-resistant MRSA	cSSSIs	II
**ME 1036**	Carbapenem	Gram-positive, including MRSA and VRE, and Gram-negatives such ESBL-strains but not P. aeruginosa	CAP	Preclinicaldevelopment
**ME1071**	Betalactamaseinhibitor	Gram-negative		I
**MK-7655**	Betalactamaseinhibitor	Gram-negative	cIAI, cUTIs	II
**Nemonoxacin**	Quinolone	Gram-positive and Gram-negative	CAP	III
**Omadacycline**	Tetracycline	Gram-positive and Gram-negative	cSSSIs, CAP	III
**Oritavancin**	Glycopeptide	Gram-positive, including MRSA, VRSA, VRE	cSSSIs	III
**Panipenem**	Carbapenem	Gram-negative and positive	cUTIs, RTI, obstetrical and gynaecological infections	III
**Plazomicin**	Aminoglycoside	MDR enterobacteriaceae and S. aureus, including aminoglycoside-resistant and metallo-ß-lactamase producers	cUTI, cIAI	II
**Radezolid**	Oxazolidinone	Gram-positive	CAP, SSTI	II
**Razupenem**	Carbapenem	Gram-negative and Gram-positive	cSSSIs	II
**Solithromycin**		Gram-positive	CAP	III
**Tebipenem/pivoxil**	Carbapenem	Gram-positive and Gram-negative	otolaryngological/RTI	II
**Tedizolidphosphate**	Oxazolidinone	Gram-positive, including MRSA	cSSSIs	III
**Telavancin**	Glycopeptide	Gram-positive	cSSTI, HAP, VAP	Approved by FDA and EMA
**Tomopenem**	Carbapenem	Gram-positive, including MRSA and Gram-negatives including ESBL-producing Enterobacteriaceae	cSSSIs, HAP	II

*NK* not known, *RTI* respiratory tract infections, *cSSTI* complicated skin and soft tissue infections, *cIAI* complicated intra-abdominal infections, *CAP* community acquired pneumonia, *HAP* hospital acquired pneumonia, *VAP* ventilator associated pneumonia, *UTI* urinary tract infections.

### Cephalosporins

The cephalosporin class of antimicrobial agents is known for its broad spectrum of activity, proven efficiency and favorable safety profile, making it the most commonly prescribed class of antimicrobials. There are four recognized class generations of cephalosporins based on their activity spectrum. Now with ceftaroline-fosamil and ceftobiprole, a new subclass of antimicrobials, cephalosporins with anti-methicillin-resistant *Staphylococcus aureus* (MRSA) activity has been introduced. Ceftaroline and ceftobiprole have also been described in the literature as ‘fifth-generation’ cephalosporin; however, such classification suggests a broader Gram-negative profile whereas ceftaroline and ceftobiprole spectrum of activity is truly unique for its expanded Gram-positive activity beyond all other presently available cephalosporins (i.e. MRSA).

**Ceftaroline fosamil** is a new, bactericidal, parental cephalosporin with expanded Gram-positive activity, including vancomycin-resistant *S. aureus* (VRSA) and MRSA. Like other beta-lactam antibiotics it works binding to penicillin-bindings-proteins (PBP) on bacterial cell walls leading to irreversible inhibition of cell-wall synthesis. The anti MRSA activity is attributed to its ability to bind PBP 2a with high affinity and to inhibit the activity of BPB 2a more efficiently than other available beta-lactams [8]. Ceftaroline has activity against Gram-positive organisms, including *S. pneumoniae*, *S. aureus,* including MRSA and *Streptococcus pyogenes*, and Gram-negative species (*Haemophilus influenzae* and *Moraxella catarrhalis*), including resistant phenotypes [9]. It has been the only Food and Drugs Administration (FDA) approved cephalosporin with activity against hetero-resistant vancomycin-intermidiate *S. aureus* (hVISA), and vancomycin-resistant *S. aureus* (VRSA) [10]. Clinical trials have demonstrated that ceftaroline was non-inferior to the standard of care for the treatment of community-acquired pneumonia (CAP) and respectively for skin and soft structure infections (SSTI). [11,12]. Ceftaroline has been approved by FDA in 2010 and by European Medical Agency (EMA) in 2012 for the treatment of acute bacterial SSSIs and CAP. Ceftaroline was reported to have synergy when combined with amikacin, tazobactam, meropenem and aztreonam. Even if several studies have demonstrated low probability of developing resistance to ceftaroline, this cephalosporin seems to induce AmpC beta-lactamases despite minimal inhibitory concentration (MIC) values in susceptible range and this is the reason why this cephalosporin should be avoided against Gram-negative bacteria known to harbor inducible Amp-C beta-lactamases [13].

The positive attributes of ceftaroline with respect to antimicrobial stewardship proGrams are: the low potential for resistance development and the favorable safety and tolerability profile in clinical trials. Ceftaroline represents now an interesting agent for the treatment of cSSSIs and CAP; future studies with intramuscular (im) formulation should enroll ceftaroline as a potential alternative to intravenous (iv) administration in outpatient setting.

### Ceftobiprole

Ceftobiprole (formerly BAL9141) is the active component of the prodrug BAL5788 (ceftobiprolemedocaril), and represents a new cephalosporin with expanded activity against Gram-positive bacteria.

Ceftobiprole was refractory to hydrolysis by the common staphylococcal PC1 beta-lactamase, the class A TEM-1 beta-lactamase, and the class C AmpC beta-lactamase, but labile to hydrolysis by class B, class D, and class A extended-spectrum beta-lactamases, thus similar to cefepime and ceftazidime patterns of action [14]. Ceftobiprole and cefepime generally had lower MICs than ceftazidime for over-expressing AmpC-producing organisms, such as *Enterobacter cloacae*. MICs for all streptococcal species, except the penicillin-resistant *Streptococcus viridans*, but including penicillin-resistant *Streptococcus pneumoniae*, ranged from < or = 0.008 to 2.0 mg/L. Ceftobiprole is active against *Enterococcus faecalis* (MIC_90_ = 4 mg/L) but generally not active against *Enterococcus faecium* (MIC_90_ > 16 mg/L). It also displayed bactericidal activity against Gram-negative pathogens comparable to cefepime, ceftazidime or piperacillin-tazobactam in early studies [15]. Recent data proved activity against *Pseudomonas aeruginosa* superior to that of cefepime [16,17]. A surveillance study on *S. pneumoniae* called, TRUST 12, showed that ceftobiprole was the most potent cephalosporin tested against *S. pneumoniae* with MIC_50_ (0,015microg/mL) and MIC_90_ (0,5microgr/mL) with values two-fold lower than ceftriaxone [17,18].

Studies of clinical isolates and strains derived from surveillance studies reported MIC_90_ values for MRSA and coagulase-negative *Staphylococcus species* of 2 to 4 and 0.5 to 8 μg/mL, respectively [19]. Ceftobiprole demonstrated an *in vivo* activity against a large sample of community acquired-MRSA clones identified in bone infections [20]. Study in healthy volunteers and in treated patients did not report the occurrence of significant adverse events [21]. The broad spectrum of activity demonstrated by ceftobiprole *in vitro* and *in vivo* suggests that it may have potential for empirical treatment of possible Gram-negative and Gram-positive infections, including those caused by MRSA.

To date, the results of three Phase III clinical trials investigating ceftobiprole for the treatment of cSSSIs and hospitalized CAP have been published [22]. In all clinical and microbiological analyses conducted Ceftobiprole was non-inferior to the agent compared to, suggesting for this new cephalosporin a potential role in treating cSSTI and hospitalized CAP patients.

Ceftobiprole has been approved for use in Canada and Switzerland, and is under review by regulatory authorities in United States, the European Union, Australia, Russia and South Africa. In November 2008 the FDA and in 2010 the EMA declined to approve ceftobiprole, citing data integrity concerns with two of the supporting studies.

**Ceftolozane/tazobactam** formerly referred to as CXA-201, is a novel oxyimino-aminothiazolyl cephalosporin (ceftolozane) and β-lactamase inhibitor (tazobactam) combination being developed for the treatment of serious Gram-negative infections [23,24]. Ceftolozane showed excellent *in vitro* activity against a panel of >900 *P.aeruginosa* strains, including cephalosporin- and carbapenem-resistant isolates. In both the US and Western Europe, Ceftolozane/Tazobactam (C/T) have proven to be active against the majority of P.aeruginosa strains whereas resistance occured in more than 20% of commonly used antibiotics (Cubist: data on file). In addition to its excellent activity against P.aeruginosa, C/T demonstrated good to excellent *in-vitro* activity against other important Gram-negative organisms, such as *E. coli* and *K. pneumoniae* (Cubist: data on file). The addition of tazobactam to ceftolozane resulted in an improved activity compared to other antibiotics such as ceftazidime.

C/T has completed phase II trials in complicated intra-abdominal (cIAI) infections (combined with metronidazole). These data have yet not been published. Phase III trials are ongoing for complicated urinary tract infection, cIAI and hospital acquired pneumonia (HAP). Ceftolozane alone has already been studied in phase II trials for complicated urinary tract infection, where it performed similarly to ceftazidime among ceftazidime-susceptible organisms [25].

Given the current *in vitro* data C/T appears as a very promising anti-Pseudomonas option.

### Carbapenems

Carbapenems have the widest spectrum of antibacterial activity of all the beta-lactams and provide excellent coverage of many Gram-negative and Gram-positive aerobic and anaerobic bacteria [26].

Similar to penicillin and cephalosporins, carbapenems are bactericidal agents that bind to the PBPs inhibiting the bacterial cell wall synthesis. They show less resistance than other beta-lactams because of their stability to hydrolysis by many extended-spectrum chromosomal and plasmid-mediated beta-lactamases, including AmpC and extended-spectrum beta-lactamases (ESBLs).

All the carbapenems already in the market or in development are reported in Table 2.

**Table 2:** Classification of carbapenems in three groups

	**Group 1**	**Group 2**	**Group 3**
Carbapenems	Ertapenem	Imipenem	Tomopenem
	Panipenem	Meropenem	Razupenem
	Tebipenem	Doripenem	
		Biapenem	
Activity against non-fermentants (*P.aeruginosa, A. baumanii*)	No	Yes	Yes
Activity vs MRSA	No	No	Yes

**Doripenem** is a carbapenem with similar properties to those of meropenem and a molecular structure that confers beta-lactamase stability and resistance to inactivation by renal dehydropeptidases [27]. Doripenem was approved by the FDA in 2007 for the treatment of pyelonephritis, complicated urinary tract infection (cUTI) and cIA. In Europe was also approved for HAP, including ventilator-associated pneumonia (VAP) [28-31].

Doripenem is highly active against methicillin-susceptible *Staphylococcus aureus*, but not effective against MRSA, *Enterococcus faecium* and vancomycin resistant enterococci (VRE). The action against *Enterococcus faecalis* is inferior to that of imipenem, but against pneumococci and other streptococci is excellent. Its MIC is slightly higher compared to meropenem versus ESBL-producing *Klebsiella pneumoniae, Proteus mirabilis, Serratia spp. Salmonella spp*. and *Shigella spp.* Against *E.coli* and *Citrobacter* it displays the same efficacy of meropenem. It has lower MICs than imipenem and meropenem versus *Pseudomonas aeruginosa* and *Acinetobacter baumannii.* It has the lowest MIC for *Burkholderia cepacia* compared to the other carbapenems.

Doripenem is effective against extended-spectrum β-lactamase or AmpC producers even if poor activity has been documented against metallo β-lactamases and class A and D serine carbapenemases [32]; it is also active, although less than meropenem, against anaerobic organisms, except *Clostridium species*[33].

When doripenem was used in association with colistin, it showed bactericidal and synergistic action against colistin resistant, carbapenemase producing *Klebsiella pneumoniae*[34].

To date, the results of five Phase III clinical trials investigating doripenem for the treatment of cIAI, urinary tract infections (UTIs) and hospital-acquired pneumonia, including ventilator-associated pneumonia have been published and demonstrated that doripenem was non inferior to the standard of care [28-31].

The most common adverse effects of doripenem were headache, insomnia, gastrointestinal distress, elevated AST and ALT and phlebitis. Seizures have been shown to occur less often than with other carbapenems.

The recommended dosage of doripenem is 500 mg i.v. every 8 hours infused over 4 hour. The dosage should be decreased in patients with moderate renal impairment [35].

Recently, the EMA has given new advice for the treatment of patients with HAP. A review of available data raises concerns that the currently approved dose of doripenem of 500 mg every 8 hours may not be enaugh to treat all patients with nosocomial pneumonia, including VAP. For the treatment of patients with impaired renal clearance or with non-fermenting Gram-negative pathogens infections, the Agency’s Committee for Medicinal Products for Human Use (CHMP) recommends to double the dosage to 1 g every 8 hours (Press Release- 22/6/2012- European Medicines Agency advises doctors treating patients with nosocomial pneumonia with Doribax).

**Panipenem** is a parenteral carbapenem launched in Japan, Korea and China for the treatment of urinary tract infections, lower respiratory tract infections, obstetrical/gynaecological and surgical infections.

It is co-administered with the betamipron, an organic anion tubular transport inhibitor that inhibits panipenem uptake into the renal tubule. Like other carbapenems, panipenem has a broad spectrum of activity covering several Gram-negative and Gram-positive aerobic and anaerobic bacteria.

Many *Enterobacteriaceae* are highly susceptible to panipenem, including *Escherichia coli, Klebsiella pneumonia, Morganella morganii, Proteus mirabilis* and C*itrobacter freundii,* it has a variable activity against *Serratia marcescens and Providencia rettgeri*, but is inactive against *Stenotrophomonas maltophilia.*

Against *Pseudomonas aeruginosa* it shows similar or less activity compared to imipenem, but less than meropenem.

It is also effective against MSSA, *S. epidermidis* and *Enterococcus faecalis*, even if, like imipenem, it has lower activity against *Enterococcus faecium* and MRSA. Panipenem proved to have a good activity against anaerobes such as *Bacteroides fragilis* and moderate activity against *Clostridium difficile*[36].

Three large trials, in patients with bacterial pneumonia, in respiratory tract infections and UTIs, have been conducted to compare panipenem/betamiprom with imipenem/cilastatin: in all three studies it displayed similar efficiency [37].

It is supplied as 1/1 g administered in two divided doses by intravenous infusion; in severe infections, the dosage may be increased to 2 g/2 g in two divided doses. The most common adverse effects observed with panipenem were skin rash, diarrhea, eosinophilia and elevation of serum hepatic transaminases. Co-administration of panipenem/betamipron and valproic acid (sodium valproate) is contraindicated, due to favoring the seizures [37].

**Biapenem** is a parenteral carbapenem that was launched in Japan in 2002 and, in USA, is currently in Phase II study. It displays excellent activity against a wide range of isolates of Gram-negative and Gram-positive anaerobes, including b-lactamase-producing strains. It also has shown a good *in vitro* activity against a broad spectrum of Gram-negative, particularly is considered to be more active than imipenem against most Enterobacteriaceae, including those producing extended-spectrum beta-lactamases. However, biapenem showed variable activity against *Serratia marcescens* (MIC90 range 0.5 to 8 mg/L) and is inactive against *Providencia rettgeri* (MIC90 > 8 mg/L). Although its *in vitro* activity against *P. aeruginosa* seems seems to be similar to that of imipenem, Biapenem is also active against Gram-positive bacteria, but not against MRSA and *E.faecium.* It shows the same moderate activity against *E.faecalis* as imipenem, meropenem and panipenem [38].

In a multicenter, randomized controlled clinical studyit was compared with imipenem/cilastatin for the treatment of respiratory and UTIs showing the same efficacy and tolerability profile [39].

This new carbapenem is supplied as 300 mg intravenously administered twice daily. The most common adverse events in comparative clinical trials were skin eruptions/rashes, nausea, vomiting and diarrhea [40].

**Razupenem** (also known as SMP-601, PTZ601, PZ-601, or SM-216601) is a new parenteral carbapenem for the treatment of complicated skin and soft-tissue infections [41]. It has a broad-spectrum activity against Gram-positive and Gram-negative pathogens, including MRSA, penicillin-resistant *Streptococcus pneumoniae*, vancomycin-resistant *E.faecium *(VREF), ampicillin-resistant *Haemophilus influenzae*, and extended-spectrum ß-lactamase (ESBL)-producing bacteria even if its activity is reduced by AmpC enzymes and carbapenemases [41,42].

**ME 1036** is an intravenous carbapenem still in clinical trials. It has *in vitro* potency against resistant Gram-positive organisms, including MRSA and VRE, and against Gram-negatives such ESBL-producing *E. coli* and *K. pneumoniae*, but it is not effective against *P. aeruginosa*[43]. Data from a 2009 study have shown its interesting utility in treating hospitalized patients with bacteraemic community acquired pneumonia (CAP) [43].

**Tomopenem** (also known as CS-023) is a new carbapenem with broad-spectrum activity against Gram-positive and Gram-negative pathogens for the treatment of cSSSI and HAP. It displays a good activity against ceftazidime-resistant *Pseudomonas aeruginosa*, ESBL-producing *Enterobacteriaceae*, penicillin-resistant *Streptococcus pneumoniae, H*. *influenzae* and MRSA, with a low rate of spontaneous resistance [44]. *In vitro* activity is comparable to that of imipenem against most Gram-positive pathogens and similar to meropenem against Gram-negative isolates even if against MRSA it shows a better activity (MIC of 4 mg/L) [44,45]. Furthermore after administration it reaches rapidly the extracellular fluid thanks to its low degree of protein binding [46].

**Tebipenem/pivoxil** (TBPM-PI, ME1211) has been under development as the first oral carbapenem for the treatment of upper respiratory tract infections. It’s not active against MBL-producing pathogens and MRSA, meanwhile is active against MDR *S. pneumoniae* and other Gram-positives, as well as Enterobacteriaceae like *K. pneumoniae* and *E. coli*[47,48].

### Monobactams

Monobactams are β-lactam compounds where in the β-lactam ring is alone and not fused to another ring (in contrast to most other β-lactams, which have at least two rings). They work only against Gram-negative bacteria.

**BAL30072 (SFM)** is a new monocyclic beta-lactam antibiotic currently in Phase I clinical testing, with potent antimicrobial activity against a broad range of Gram-negative bacteria. It is a siderophore-monobactam with potent *in vitro* activity against MDR Gram-negative bacilli, representing an interesting option in treating carbapenem-resistant *A. Baumanii* isolates [49,50]. Actually it is stable against most carbapenemases including KPC, metallobeta-lactamases, including the New Delhi metallo-beta-lactamase 1 (NDM-1), and the class D carbapenemases, mostly found in *Acinetobacter spp.*[51].

In a recent study Hofer and colleagues demonstrated that BAL30072 in association with carbapenem has a synergic action against *P. aeruginosa* and *Enterobacteriaceae* in interfering with resistance development, particularly in strains with inducible cephalosporinases [51].

### β-lactamase inhibitors

In β-lactam agent/β-lactamase inhibitor combinations, the latter potentiates the action of the former by protecting it from enzymatic hydrolysis. Currently used β-lactam/β-lactamase inhibitor compounds are highly active against class A and various ESBLs, but with poor activity against class C and class D enzymes.

Several compounds are now under investigation as potential β-lactamases inhibitors, in different stages of pre-clinical and clinical studies. They can be classified according to their molecular structure as β-lactams and non-β-lactams. Their main advantage over the older available β-lactamase inhibitors is conferred by the ability to inhibit class C and D enzymes. Thus, MIC of various currently used β-lactams, such as piperacillin or ceftazidime, is decreased when administered together with novel β-lactam inhibitors, these antibiotics become active against ESBL-producing strains. Moreover, used combined with carbapenems, makes the latter active against MBL-producing strains.

Even though, the results of studies on clinical usefulness of new β-lactam inhibitors are not available yet, they seem particularly promising as therapeutic agents.

**Avibactam** (also known as **NXL104)** is a β-lactamase inhibitor that has no antibacterial activity, but has interesting property to inhibit beta-lactamases. Currently it is in clinical development combined with both ceftazidime and ceftaroline. It displays a broad-spectrum inhibitory profile against enzymes belonging to classes A and C β-lactamases (including AmpCs, ESBLs, and KPC) [52], on the other side, in combination with aztreonam it offers a potential option against bacteria producing NDM-1 [53].

There are several on-going studies in phase III, assessing the efficacy in association with ceftazidime in the treatment of cIAI, HAP and cUTI [54,55].

**MK-7655** is a novel beta-lactamase inhibitor under clinical development. It displays good *in vitro* activity against class A and class C carbapenemases, especially when combined with imipenem/cilastatin. Currently the drug is in phase II clinical development trial for the treatment of cIAI and cUTI [56].

**ME1071 (CP3242),** a class-B inhibitor, maleic acid derivative, is a novel specific inhibitor for metallo-β-lactamases (MBL). It reduces the MICs of carbapenems for bacteria with NDM-1 enzyme. It can potentiate the activity of carbapenems (expecially biapenem) and ceftazidime against MBL-producing strains of *P. aeruginosa*and other Gram-negative bacteria, as *E. coli*, *Serratia marcescens*, *A. Baumanii* and *K. pneumoniae.* It shows less activity against bacteria with IMP and VIM metallo-enzymes [57].

### Aminoglycosides

The aminoglycoside class of antibiotics consists of many different agents. As an example, nine (gentamicin, tobramycin, amikacin, streptomycin, neomycin, kanamycin, paromomycin, netilmicin, and spectinomycin) are approved by the FDA and EMA for clinical use in the United States and Europe. Of these, gentamicin, tobramycin, and amikacin are the most frequently prescribed, although netilmicin possesses comparable efficacy for selected indications.

The most common clinical indiation (either alone or as part of combination therapy) of the aminoglycosides is the treatment of serious infections caused by aerobic Gram-negative bacilli. While less common, aminoglycosides (in combination with beta-lactams) have also been used for the treatment of select staphylococcal and enterococcal infections.

Relative to other classes of antibiotics, the aminoglycosides are have demonstrated relative stability against the development of resistance. Treatment-emergent resistance (especially when used in combination with other agents) is rare.

**Plazomicin (ACHN-490)**is an aminoglycoside with a bactericidal dose-depending activity that inhibits bacterial protein synthesis. This new intravenous aminoglycoside demonstrates activity against Gram-positive and Gram-negative pathogens [58]. It showed an *in vitro* synergism with daptomycin and ceftobiprole against MRSA, hVISA and VISA and with doripenem, imipenem, piperacillin/tazobactam and cefepime against *P. aeruginosa*[59].

Phase II study in patients with cUTI and acute pyelonephritis, including cases with concurrent bacteremia, compared plazomicin with levofloxacin [60].

### Quinolones

The quinolone class of antimicrobial agents has generated considerable interest since its discovery >40 years ago. Substantial progress has been made in our understanding the molecular mechanisms of action of quinolones against pathogenic bacteria, the induction of resistance to quinolones in these organisms, and the potential of each quinolone compound to induce toxicity in treated patients. A number of infectious diseases are successfully treated with quinolones administered orally or intravenously, but nowadays its clinical utility is diminished due to the widespread of quinolone resistance, especially in Gram-negative rods. The future of the quinolones is difficult to predict. Nevertheless, the quinolone nucleus continues to provide opportunities for future modifications that may produce more valuable compounds.

**Besifloxacin** is a topical ophthalmic fluoroquinolone, approved by the FDA in May 2009 to treat bacterial conjunctivitis caused by susceptible bacterial strains. It is active against most common ocular bacterial pathogens, including *Staphylococcus aureus*, *Streptococcus pneumoniae*, and *Haemophilus influenzae. A*mong MDR pathogens such as vancomycin resistant (VR) *Enterococcus faecalis* and *E.faecium*, ciprofloxacin-susceptible MRSA, and ciprofloxacin-resistant MRSA, MIC_90_ values were lower than that of other fluoroquinolones [61].

**Nemonoxacin (TG-873870)** is a novel non-fluorinated quinolone in clinical development (phase 3), that displays a potent *in vitro* and *in vivo* activity against CAP pathogens. It has a broad-spectrum antibacterial activity, higher than levofloxacin, against both Gram-positive (*S. aureus, S. capitis, S. pneumoniae and E. faecalis)*, and Gram-negative bacteria (*E. coli*) isolates.

It has also demonstrated a potent antibacterial activity against ciprofloxacin-resistant MRSA, methicillin- and levofloxacin-resistant *Staphylococcus capitis*, penicillin and levofloxacin-resistant *S. pneumoniae*and VRE [62].

It has similar activity to ciprofloxacin, moxifloxacin and levofloxacin against *Enterobacteriaceae*. Against *P. Aeruginosa* nemonoxacin has similar activity to moxifloxacin, and the activity against *S. maltophilia* is comparable to levofloxacin. Oral nemonoxacin (750 mg and 500 mg) administered for seven days showed similar clinical and bacteriological response as levofloxacin in the therapy of CAP [63,64].

**Zabofloxacin (DW-224a)** is a novel fluoroquinolone antibiotic agent, currently in phase II development in USA. The study which has compared zabafloxacin with levofloxacin in the treatment of CAP has not been published yet. In the preliminary results of a double-blind, randomized, multicenter study in South Korea, the new quinolone displayed the same clinical and microbiological results as moxifloxacin in adult patients with mild-to-moderate CAP. It could be an alternative in treating acute bacterial exacerbation in chronic obstructive pulmonary disease (COPD) [65].

**Delafloxacin (RX-3341)** is an investigational oral and parental fluoroquinolone active against a variety of Gram-positive bacteria, including methicillin- and quinolone-resistant strains of *Staphylococcus aureus* (MRSA, QRSA). It presents also an interesting activity against quinolone-resistant strains of *Pseudomonas aeruginosa* or *Klebsiella pneumoniae*[66,67].

**JNJ-Q2** is a fluorinated 4-quinolone developed for the treatment of acute bacterial SSTI and respiratory tract infections. In a recent study involving 118 isolates of *Streptococcus pneumoniae*, including fluoroquinolone-resistant variants, it was 32-fold more potent than moxifloxacin. It has also shown a better action than moxifloxacin against MRSA. The activity of JNJ-Q2 against Gram-negative pathogens is generally comparable to those of moxifloxacin. Rates of spontaneous development of resistance to JNJ-Q2 in *S. pneumoniae*, MRSA, and *Escherichia coli* were indicative of a lower potential for resistance selection than other fluoroquinolones [68].

### Oxazolidinones

The oxazolidinones are a new class of antimicrobial agents which have a unique structure and good activity against Gram-positive pathogenic bacteria. Oxazolidinones are a class of compounds containing 2-oxazolidine in the structure. Oxazolidinones represent a new class of synthetic antibacterial agents active against multiple-resistant Gram-positive pathogens, including MRSA, penicillin-resistant streptococci, and VRE.

Oxazolidinones inhibit protein synthesis by binding to the P site at the ribosomal 50S subunit. Resistance to other protein synthesis inhibitors does not affect oxazolidinone activity, however rare development of oxazolidinone resistance cases, associated with 23S rRNA alterations, during treatment have been reported. Linezolid, the first and at the moment the only oxazolidinone available, has already taken its place in the clinical setting for the treatment of Gram-positive infections. Pharmacokinetic properties as well as its good penetration and accumulation in the tissue including skin, bone, lung, vegetations, haematoma and cerebrospinal fluid, allow its use for several type of infections.

**Tedizolid phosphate (TR-701),** previously known as torezolidphosphate, is an inactive prodrug that, after oral or intravenous administration, is rapidly converted to the active form (torezolid). Tedizolid is a new oxazolidinone designed for the treatment of infections caused by Gram-positive bacteria with resistance to penicillin and other antimicrobial classes; it showed an improved antibacterial efficacy, especially against linezolid-resistant strains [69]. Tedizolid has high bioavailability, penetration, and tissue distribution when administered orally or intravenously. The activity of tedizolid was better than linezolid’s against strains of *Staphylococcus spp., Streptococcus spp., and Enterococcus spp. in vitro* studies, including strains resistant to linezolid and those not susceptible to vancomycin or daptomycin. Choi and collegues showed that tedizolid is four-fold more potent *in vitro* than linezolid against *S. pnemoniae* penicillin resistant [70]. Its pharmacokinetic characteristics allow an once-daily administration that leads to a more predictable efficacy and safety profile than those of linezolid. No hematological adverse effects have been reported associated with tedizolid when used at the therapeutic dose of 200 mg in Phase I, II, or III clinical trials of up to 3 weeks of tedizolid administration. Given that the clinical and microbiological efficacy are similar for the 200, 300, and 400 mg doses, the lowest effective dose of 200 mg once daily for 6 days was selected for Phase III studies in acute bacterial skin and skin-structure infections, providing a safe dosing regimen with low potential of developing myelosuppression. Unlike linezolid, tedizolid does not inhibit monoamine oxidase *in vivo*, therefore interactions with adrenergic, dopaminergic, and serotonergic drugs are not to be expected [71].

Tedizolid has been compared with linezolid in a phase III study in the treatment of acute bacterial SSTI. A short (6-day) course of tedizolid was as effective as a 10-day course of linezolid with regard to both early and sustained clinical responses [72].

### Radezolid (RX-1741)

The goals of the development proGram of this new oxazolidinone included expansion of the spectrum to include fastidious Gram-negative bacteria that would facilitate empirical treatment of community-acquired pneumonia, as well as optimization of drug-like properties. The antimicrobial activity of radezolid was evaluated against respiratory pathogens demonstrating significant better activity than linezolid with anMIC_90_ of 0.25 mcg/mL against both *S. Pneumonia* and *S. pyogenes*. MIC_90_ values for staphylococci ranged from 1–4 mcg/mL and 0.5–1 mcg/mL for enterococci [73]. Radezolid was clearly differentiated from linezolid and the other oxazolidinones versus *Haemophilus influenzae* and *Moraxella catarrhalis*, with an MIC_90_ of 1 mcg/mL and 0.5 mcgg/mL, respectively [72]. Radezolid has outperformed linezolid in an *E. faecium*VanA peritonitis model when dosed [74].

Radezolid was selected for further advancement with two phase 2 clinical trials completed to date: the first in CAP and the second trial in cSSSI [75].

Radezolid offers the potential for several incremental improvements in that it is generally two-fold more active *in vitro* than linezolid against the staphylococci and 4- to 16-fold more potent against the streptococci and enterococci. Unique for the oxazolidinones, radezolid offers coverage of the fastidious Gram-negative organisms. It is unclear at this point, based upon published literature, whether radezolid has any appreciable safety advantages over linezolid. To date, phase III trials have not been initiated.

### Glycopeptides

Glycopeptide antibiotic are large, rigid molecules that inhibit a late stage in bacterial cell wall peptidoglycan synthesis. They bind to the amino acids within the cell wall preventing the addition of new units to the peptidoglycan. In particular, they bind to acyl-D-alanyl-D-alanine in peptidoglycan.

These antibiotics are effective mainly against Gram-positive cocci. They exhibit a narrow spectrum of action. Some tissues are not penetrated very well by old glycopeptides and they do not penetrate into the cerebrospinal fluid. Several derivates of vancomycin are currently being developed, including oritavancin and dalbavancin (both lipoglycopeptides). Possessing longer half-lives than vancomycin, these newer candidates may demonstrate improvements over vancomycin due to less frequent dosing and activity against vancomycin-resistant bacteria.

**Oritavancin** is a semisyntetic lipoglycopeptide analogue of vancomycin with at least 3 mechanisms of actions:inhibition of transglycosylation, inhibition of transpeptidation and cell memebrane distruption/interaction [76,77]. Oritavancin seems promising for the treatment of serious infections caused by Gram-positive bacteria, such as MRSA, VISA, VRSA, daptomycin-nonsusceptible *S. aureus* and VRE. Recent data collected in Western Europe confirm the potent *in vitro* activity of oritavancin against a wide range of resistant MRSA, MRCoNS and VRE isolates [76]. Oritavancin present a rapid concentration-dependent bactericidal activity. The pharmacodynamic (PD) and pharmacokinetic (PK) profiles of oritavancin are unique and suggest that oritavancin could be effective given in a single dose. A humanized dosing regimen mimicking a 1,200-mg single dose of oritavancin administered to neutropenic mice with *S. aureus* thigh infections resulted in a greater rate and spread of bacterial kill than did a regimen simulating 400 mg once daily for 3 days, indicating that a front-loaded dose of oritavancin could provide for faster and more sustained bacterial killing activity than an equivalent cumulative dose administered in a fractionated manner [78]. Oritavancin is not metabolized following i.v. dosing. Instead, it is slowly excreted, unchanged, in both urine and the feces (terminal half-life = 393 ± 73.5 h), which means that no dosage adjustment is required for age, or for renal or mild to moderate hepatic dysfunction.

In two phase 3 studies evaluating the efficacy of oritavancin in treating cSSSI when dosed daily for 3 to 7 days oritavancin was non-inferior to the agent compared to.

The SIMPLIFI study was designed to evaluate the non-inferiority of two front-loaded treatment regimens (a single dose and an infrequent dose on day 1with an optional dose on day 5) to the daily-dose regimen used in the previous phase 3 studies for the treatment of cSSSI due to Gram-positive pathogens. The results of this study show that single- and infrequent-dosing schedules of oritavancin were as efficient as daily administration and had a similar safety profile in treating cSSSI caused by Gram-positive pathogens, including MRSA [79,80].

**Telavancin** is a vancomycin-derived lipoglycopeptide administered once-daily and characterized by a broad-spectrum of microbiologic activity against Gram-positive bacteria. Telavancin has a fast bactericidal activity and multiple mechanisms of action. Telavancin exhibits potent *in vitro* antibacterial activity against a broad range of clinically important Gram-positive bacteria, including MRSA. The unique structure of telavancin, which is derived from vancomycin with the addition of a hydrophobic side chain and a hydrophilic group (precisely a glycolipopeptidic structure), is responsible for the improved activity against isolates with reduced glycopeptide susceptibility [81,82].

The excellent activity of telavancin against Staphylococcus spp. represents the main characteristic of this compound. MIC values (MIC_90_) of tested strains between 0.25 and 1 mg/L have been reported in an over 4,500 isolates of methicillin-susceptible *S. aureus* (MSSA) and MRSA worldwide 11–17. In several studies, telavancin MICs for MRSA ranged from two to eight times lower than those observed for vancomycin, teicoplanin, and linezolid [83]. Similarly, coagulase-negative staphylococci (CoNS) exhibited MIC_90_ values between 0.25 and 1 mg/L. Telavancin also proved to have an excellent activity against MRSA and CoNS with reduced susceptibility to glycopeptides, together with both vancomycin-susceptible and resistant enterococci [83]. The potent anti-staphylococcal activity of telavancin may lead to consider this lipoglycopeptide as an alternative to vancomycin in cases of difficult-to-treat MRSA infections [84,85]. In fact telavancin has been associated with a ten-fold greater peptydoglican synthesis inhibitory activity against MRSA than vancomycin[86]. Two randomized clinical trials demonstrated the efficacy and safety of telavancin compared to vancomicin in the treatment of nosocomial pneumonia [87,88].

Penetration into skin blister fluid was approximately 40% of plasma levels, but was sufficient to eradicate pathogens which might be present. Telavancin penetrates the pulmonary epithelial lining fluid and alveolar macrophages, with concentrations considerably higher in the latter. Unlike daptomycin, the *in vitro* activity of telavancin was found to be unaffected by pulmonary surfactant [83]. Given the excellent activity against MRSA and other difficult Gram-positive bacteria, telavancin seemed appropriate for treating complicated skin and skin structure infections (cSSSI). Results from phase 2 and 3 clinical trials with telavancin for cSSSI have been published and have demonstrated similar efficacy and tolerability compared to standard anti-staphylococcal beta-lactams and vancomycin.

Telavancin has been also evaluated in two studies in the treatment of HAP due to Gram-positive cocci, particularly MRSA. The results of these two trials demonstrated that telavancin has clinical response outcomes that are non-inferior to those of vancomycin. More important, these findings, which incorporate data for more than 1500 patients from >250 sites around the world, are robust and consistent across all efficacy populations. In patients with pre-existing moderate/severe renal impairment (CrCl < 50 mL/min) telavancin presented an increased mortality compare to vancomycin. [89]. Telavancin is usually well tolerated, with the most commonly experienced side effect being gastrointestinal discomfort, but must be considered the potential elevation of serum creatinine [90].

Telavancin has been approved by FDA for the treatment of cSSSIs and by EMA for the treatment of adult with nosocomial pneumonia including VAP, suspected or known to be caused by MRSA, only in situations where it is known or suspected that other alternatives are not suitable.

Considering the limited therapeutic options for the treatment of nosocomial pneumonia due to MRSA, telavancin should represent a good alternative to standard therapy.

**Dalbavancin** is a new lipoglycopeptide that inhibits cell wall synthesis in Gram-positive bacteria through the formation of a stable complex between its heptapeptide backbone and the D-Ala-D-Ala portion of cell wall precursors. Dalbavancin demonstrates *in vitro* activity against clinically significant Gram-positive pathogens, including MSSA, MRSA, meticillin-susceptible S. epidermidis (MSSE), meticillin-resistant S. epidermidis (MRSE) and enterococci, but lacks activity against VanA-type enterococci [91]. Dalbavancin MICs for *Staphylococcus spp.* are significantly lower than that of vancomycin. Dalbavancin has been reported to be active against VISA, but shows poor activity versus VRSA [92-94]. The activity of dalbavancin against Clostridium spp. is comparable with vancomycin [95].

Like other glycopeptides, dalbavancin is poorly absorbed when administered orally, necessitating intravenous administration. The standard dose of dalbavancin is 1000 mg administered on day 1 and 500 mg administered 1 week later. The main PK characteristic is the long-half-life: the terminal elimination half-life of dalbavancin ranges from 147 to 258 hours, allowing the once-weekly dosing that maintains the serum plasma concentrations of dalbavancin above the MIC of common pathogens for 7 days [96]. The long-half life of dalbavancin is a result of its extensive protein binding , as well as its retention within cells.

Two randomized phase III studies, one already published and one not yet published, showed that dalbavancin achieved its primary endpoint of non-inferiority in acute bacterial SSTI. Researchers compared two intravenous doses of dalbavancin given one week apart with twice-daily vancomycin and linezolid doses. [97].

### Polymixin

Polymyxins are antibiotics, with a general structure consisting of a cyclic peptide with a long hydrophobic tail. They disrupt the structure of the bacterial cell membrane by interacting with its phospholipids. They are produced by non-ribosomal peptide synthetase systems in Gram-positive bacteria such as Paenibacillus polymyxa and are selectively toxic for Gram-negative bacteria due to their specificity for the lipopolysaccharide molecule that exists within many Gram-negative outer membranes.

The global problem of advancing antimicrobial resistance has recently led to a renewed interest in their use.

**CB-182,804** is a novel polymyxin analogue. It has a good *in vitro* activity against MDR Gram-negative bacteria, such as A*. baumannii, E. coli, K. pneumoniae* and *P. aeruginosa*. CB-182,804 has showed high activity against colistin-susceptible and -resistant isolates. Colistin-resistant strains that are resistant to all available antibiotics are found to be susceptible, even if a cross resistance with colistin has been observed [98]. Currently the drug is in Phase I clinical stage.

### Tetracycline

Tetracyclines are a group of broad-spectrum antibiotics whose general usefulness has been reduced with the onset of bacterial resistance. Despite this, they remain the treatment of choice for some specific indications. Tetracyclines are generally used in the treatment of urinary tract and intestinal infections, and are used in the treatment of chlamydial infections, especially in patients allergic to β-lactams and macrolides; however, their use for these indications is less popular than it once was due to widespread development of resistance in the causative organisms.

**Eravacycline (TP-434)** is a novel fluorocycline parenteral/oral antibiotic, similar to tigecycline, with a broad spectrum activity against all Gram-negative bacteria. It is active *in vitro* against MDR aerobic and anaerobic Gram-negative and Gram-positive bacteria, including emerging Gram-negative pathogens like *Acinetobacter baumanii* (MIC_90_, 2 mcg/mL) and clinically important species of Enterobacteriaceae (including E. coli [MIC_90_, 0.5 mcg/mL] or *K. pneumoniae* isolates [MIC_90_, 1 mcg/mL] that produce extended spectrum beta-lactamases [ESBL] and/or are carbapenem resistant. The drug has low potency against *Pseudomonas aeruginosa* (MIC_90_, 16 mcg/mL), but it has no known antagonism with other antibiotics that are expected to be active against this species.

The spectrum includes MSSA/MRSA, MS/R *S. epidermidis*, all the streptococci and enterococci, several anaerobes, including *Bacteroides spp.* and *Clostridium difficile*[99]*.* The mean half-life of everacycline in plasma was 47.7 hours whereas the median value was 35.3 hours. Oral bioavailability would provide an important dosing advantage for a tetracycline that has activity against MDR Gram-negative bacteria. The oral bioavailability in humans averaged about 28% for doses of 50, 100, 200, and 300 mg and oral doses were well-tolerated. Two IV doses of 1.5 mg/kg q24h and1.0 mg/kg q12h, are currently being evaluated in a phase 2 randomized, double-blind, global study for the treatment of adult cIAI [100,101].

### Omadacycline (PTK796)

Omadacycline, an aminomethylcycline, is a semisynthetic derivative of minocycline that has *in vitro* potency against Gram-positive and Gram-negative bacteria. It presents activity against MSSA/MRSA, MS or MR coagulase-negative staphylococci, *Enterococcus faecalis, Enterococcus faecium, S. pneumonia, Klebsiella pneumoniae, Proteus mirabilis/vulgaris, Providencia rettgeri/stuartii, Morganella morganii and Bacteroides fragilis.* The potency of omadacycline was not impaired by lung surfactant [102].

In a phase 2 study of over 200 adults with SSSI initially requiring IV therapy, patients were randomized to receive 100 mg q24h IV with 200 mg oral step-down dosing or linezolid at 600 mgq12h IV with a 600 mg q12h oral step-down dose. Omadacycline showed comparable efficacy across all the study populations [102].

Omadacycline started enrollment in a phase 3 ABSSSI trial in 2009 with 200 mg IV, with potential to step-down to a 150 mg oral tablet, but the trial was stopped to change their endpoints in the protocol to be as required by the new FDA guidance for trial on cSSTI.

### Pleuromutilin compound

Pleuromutilins were discovered as natural-product antibiotics in 1950. Tiamulin was the first pleuromutilin compound to be approved for veterinary use in 1979, followed by valnemulin in 1999. It was not until 2007 that retapamulin became the first pleuromutilin approved for use in humans. However, retapamulin is limited to topical application. Recent advances in lead optimization have led to the synthesis of pleuromutilins that combine potent antibacterial activity with favorable pharmaceutical properties, making these compounds suitable for oral and intravenous delivery. Most pleuromutilins have an antibacterial spectrum that covers the common pathogens involved in both skin and respiratory tract infections.

**BC-3781** is a novel semisynthetic systemic agent belonging to the pleuromutilin class of antibiotics. Pleuromutilins interferes with bacterial protein synthesis via a specific interaction with the 23S rRNA of the 50S bacterial ribosome subunit. *In vitro*, BC-3781 demonstrates clinically relevant activity against the most frequently identified Gram-positive skin pathogens, including MSSA and MRSA, *S. pyogenes, Streptococcus agalactiae*, and vancomycin-resistant *Enterococcus faecium.* Furthermore, BC-3781 exhibits significant *in vitro* activity against coagulase-negative Staphylococcus spp. and communitary respiratory pathogens, including *Streptococcus pneumoniae, Haemophilusinfluenzae, Moraxella catarrhalis, Legionella pneumophila, Chlamydophila pneumoniae,* and *Mycoplasma pneumoniae*[103,104] So far the therapeutic administration of pleuromutilin antibiotics has been limited to veterinary medicine or to the topical route in humans, due to associated toxicities.

Recently the phase 2 study in the treatment of cSSTI caused by a Gram-positive pathogen has been completed by comparing BC-3781 to vancomycin. The results provided the first proof of concept for the systemic use of a pleuromutilin antibiotic in the treatment of SSSIs [105].

## Conclusions

The creation of new antibiotics targeting the growing threat of multidrug resistance is a goal that remains "alarmingly elusive”. In a recent article, the IDSA reports that only a single new antibiotic has been approved by FDA since 2010, and few new drugs are in the pipeline. The report found also that only 7 new antibiotics targeting MDR Gram-negative bacilli have reached phase 2 or phase 3 trials since 2010, when IDSA established its 10 × '20 initiative, with the goal of developing 10 new antibiotics by 2020 [106].

During the early history of antibiotics, especially in the 70′s and the 80′s, the ability to find new compounds and/or modify structural forms to extend the spectrum against resistant strains was possible and is still being pursued today. Low returns on investments and an unpredictable and often infeasible approval pathway at regulatory agencies have caused many companies to leave the antibiotics market. Resistance is the driver for new antibiotics and the incidence of MDR pathogens is ever increasing, despite the attempts of antibacterial stewardship and stringent effort to infection-control of MDR bacteria in hospital. The bad hospital pathogens have escaped the hospital and are joining the ranks of the community pathogens.

There is a number of promising antibiotics in development, regulatory approvals are crucial over the next five years to return us to a time when reliably effective treatment of bacterial diseases is again a reality, not just a future prospect.

## Competing interests

MB serves on scientific advisory boards for Astra Zeneca, Bayer, Cubist, Trius, Gilead, Pfizer Inc, Merck Serono, and AstellasPharma Inc.; has received funding for travel or speaker honoraria from Astra Zeneca, Gilead, Pfizer Inc., Merck Serono, Novartis, Gilead Sciences, Sumimoto, TevaInc and AstellasPharma Inc. The other authors declare no conflicts of interest.

## Authors’ contributions

MB have made substantial contributions to conception, design and interpretation of data; MM, CT and AA have been involved in drafting the manuscript or revising it critically for important intellectual content. All authors read and approved the final manuscript.
